# Avian Eggshell Membrane as a Novel Biomaterial: A Review

**DOI:** 10.3390/foods10092178

**Published:** 2021-09-14

**Authors:** Yaning Shi, Kai Zhou, Dandan Li, Vincent Guyonnet, Maxwell T. Hincke, Yoshinori Mine

**Affiliations:** 1 College of Food Science and Technology, Nanjing Agricultural University, Nanjing 210095, China; 9191810122@njau.edu.cn (K.Z.); lidandan@njau.edu.cn (D.L.); 2 FFI Consulting Ltd., 2488 Lyn Road, Brockville, ON K6V 5T3, Canada; vincent.guyonnet18@gmail.com; 3 Department of Cellular and Molecular Medicine, University of Ottawa, 75 Laurier Ave. E, Ottawa, ON K1N 6N5, Canada; mhincke@uottawa.ca; 4 Department of Food Science, University of Guelph, 50 Stone Road East, Guelph, ON N1G 2W1, Canada

**Keywords:** eggshell membrane, biomaterial, peptides, bioactives, human health

## Abstract

The eggshell membrane (ESM), mainly composed of collagen-like proteins, is readily available as a waste product of the egg industry. As a novel biomaterial, ESM is attractive for its applications in the nutraceutical, cosmetic, and pharmaceutical fields. This review provides the main information about the structure and chemical composition of the ESM as well as some approaches for its isolation and solubilization. In addition, the review focuses on the role and performance of bioactive ESM-derived products in various applications, while a detailed literature survey is provided. The evaluation of the safety of ESM is also summarized. Finally, new perspectives regarding the potential of ESM as a novel biomaterial in various engineering fields are discussed. This review provides promising future directions for comprehensive application of ESM.

## 1. Structure and Chemical Composition of the Eggshell and Eggshell Membrane

**Figure 1:**
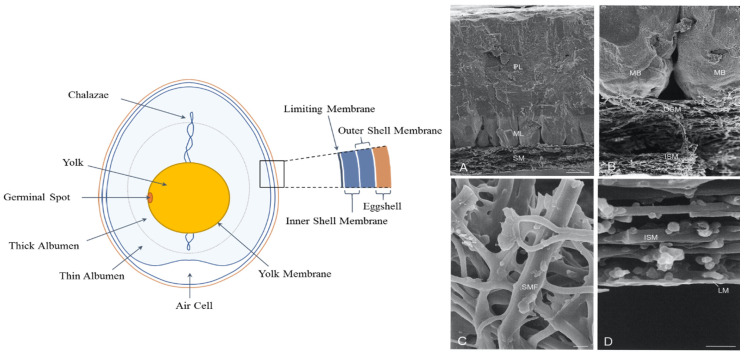
Hen egg structure and scanning electron micrographs illustrating the morphology of the eggshell and eggshell membranes. (**A**) Eggshell cross-fractured to reveal the shell membrane (SM), mammillary layer (ML), and palisade layer (PL); (**B**) higher magnification of the membrane mammillary body interface: Outer shell membrane fibers (OSM); insert into the tips of the mammillary bodies (MB); inner shell membranes (ISM); (**C**) enlargement of the shell membrane fibers (SMF), revealing their interwoven and coalescing nature; (**D**) inner aspect of the inner shell membrane (ISM), demonstrating the limiting membrane (LM) that surrounds the egg white here removed during sample preparation. Scale bars: (**A**), 50 mm; (**B**), 20 mm; (**C**,**D**), 2 mm. (adapted from M.T. Hincke et al., Matrix Biology, 19, 443–453, 2000, [5]).

The avian eggshell, representing about 10% of the egg weight, is composed of the shell and shell membrane. The shell is a calcareous structure predominantly constituted of calcium carbonate (CaCO_3_) (95%) and an organic matrix composed of proteins, glycoproteins, and proteoglycans (3.5%) [1,2]. The eggshell membrane (ESM) consists of cross-linked collagens (I, V, and X), glycosaminoglycans (GAGs), egg white proteins (i.e., Ovotransferrin, Lysozyme) and eggshell matrix proteins (i.e., Ovocalyxin-36) [1,2,3,4,5]. The ESM is the innermost component of the eggshell, lying in between the mammillary layer and the egg white. It features a unique fibrous net structure, allowing the mineralization process of the eggshell from the outer surface of the ESM, as well as keeping the egg white from mineralization [6,7]. During the early stage of incubation, the ESM is firmly combined to the mammillary cones, and it is difficult to separate through mechanical action. However, this connection weakens as incubation progresses [8]. The ESM is divided into three layers: the outer shell membrane, the inner shell membrane, and the limiting membrane. The detailed structure of the egg shell and ESM is indicated in Figure 1, showing the triple-layer structure with a spiral arrangement of these layers [9].

The outer shell membrane represents the outmost layer of the ESM and facilitates the close attachment to the eggshell. Fibers in the outer shell membrane present bud-like structures on top of the mammillary knob, allowing a strong binding between the ESM and the eggshell [8]. The outer shell layer is also the thickest of the three layers, with a thickness of approximately 50–70 μm [1,10]. The fibers in the inner shell layer intertwine with fibers in the outer shell membrane, except in the air cell region [11]. The limiting layer is a slender structure that directly covers the egg white [7]. Due to the presence of a great number of fiber knobs, the outer shell membrane is rougher than the inner shell membrane [12]. In addition, the fibers in the three layers of the ESM vary in diameter, decreasing from the outermost to the limiting membrane [13].

The ESM is rich in protein-based fibers, comprising of about 80–85% proteins [14]. It was reported that the ESM contains over 500 different types of proteins [15]. Collagens are the major structural basis of the fibers, making up 10% of the ESM [16]. The ratio of collagen I and V is about 100:1, with their contents in the outer and inner layers of the ESM significantly different [17]. While the inner ESM contains both collagen I and collagen V, the outer ESM presents only collagen I [18]. Another type of collagen, collagen X, is found in both the outer and inner layers of the ESM. Collagen X is believed to inhibit the mineralization process, preventing both the egg white and yolk from mineralization [7,19]. However, such a hypothesis is in contradiction with the fact that collagen X is located in the core of the fibers [6]. Fibronectin, a dimeric form glycoprotein with the function of activating or binding proteins, is another type of protein present in the ESM [20]. Osteopontin, which contains numerous binding sites for cell and calcium as well as various serine/threonine phosphorylation sites is also present in the ESM [21]. In addition to these various proteins, CaCO_3_ minerals are also present in the ESM, along with sialic acid, uronic acid, and small amounts of saccharides [3,22]. The main chemical components of the eggshell membrane and their functions are summarized in Table 1.

## 2. Isolation and Solubilization of the Eggshell Membrane

Generally, the ESM can be separated from the eggshell through mechanical, chemical, or enzymatical treatment. The inner ESM and limiting membrane are embedded into the ES; hence, they must be isolated manually [11]. However, the fibers in the outer ESM are firmly combined to the mammillary cones in the eggshell, and the separation requires additional operations [3]. Since CaCO_3_ is the major component of the eggshell, its dissolution under acidic condition will break the strongly bounded structure and release the ESM [13,51,52]. Acid treatment with acetic acid, hydrochloric acid, or EDTA is commonly applied for the separation of the ESM [53,54,55,56]. Another strategy consists of loosening the bounds between the ESM and ES by immersing the ES in low acid solution followed by manual separation [54,55,57,58]. During the acid treatment process, numerous parameters such as the incubation temperature, reaction time, moisture content, and type of acid used will influence the efficiency of this separation step [56,59].

The ESM is hard to dissolve in aqueous solution due to the various interactions between CREMPs [60], keratins, desmosines, and hydroxylysinonorleucine [61,62], impeding its processing and application. However, the preparation of eggshell membrane protein (SEP) in water-soluble solutions would strongly facilitate their utilization and maximize the economic potential of ESM. Due to the protein composition of the ESM, the temperature applied during the preparation of SEP must be controlled to avoid the denaturation of collagens and other fibrous proteins (50–70 °C) [63,64]. In previous studies, scientists treated the ESM for reductive cleavage with aqueous 3-mercaptopropionic acid and acetic acid [65]. The bioactivity of the SEP recovered by this technique was confirmed by growing NIH3T3 cells in the presence of SEP. Additional investigations are conducted to improve the suitability of SEP to specific practical applications, including the extraction of specific ESM proteins. Jia et al. developed SEP/PLGA electrospun nanofibers, which can prevent the invasion of epithelial tissue and provides enhanced cell attachment conditions, making them an ideal biomaterial for tissue regeneration [66]. Zhang et al. have combined acetic acid decalcification, EDTA decalcification, and phosphate buffer extraction to purify efficiently the eggshell matrix proteins OC-17, OC-116, and OCX-36 [59]. Response surface methodology is used for the extraction of pepsin-soluble collagen [67]. According to the results, the optimum extraction conditions included alkali hydrolysis with 0.76 mol/L NaOH for 18 h and enzymatical hydrolysis with 50 U/mg pepsin for 43.42 h. The extraction yield was 30.0% [67]. Shi et al. dissolved the ESM in alkaline 10% alcohol solution at 70 °C and recovered soluble proteins with antioxidant activity [68]. Therefore, various approaches for the processing of the ESM and the preparation of SEP have made possible to find applications for the ESM in a wide range of fields.

## 3. Application of Eggshell Membrane as a Novel Biomaterial

### 3.1. ESM for Joint Health

Osteoarthritis (OA) is the most prevalent chronic joint disease, affecting significantly the patients’ ability to function and quality of life. Very often, patients rely on dietary supplements for pain relief. The eggshell membrane has been tested as a natural therapeutic for joint and connective tissue disorders and was reported to exert some beneficial effects on joint pain, stiffness, and cartilage turnover induced by overexercise [69]. In a randomized study, postmenopausal women were assigned to a placebo and intervention group, with thirty women taking orally a commercial product, Natural Eggshell Membrane (NEM^®^), 500 mg once per day for 2 weeks while conducting regular exercise on alternate days. The results showed that the cartilage turnover was significantly reduced in the intervention group. The consumption of the eggshell membrane product rapidly improved the recovery from exercise-induced joint pain and stiffness as well as reduced the discomfort immediately after exercise [69].

A double-blinded, placebo-controlled clinical trial was conducted to study the therapeutic effect and safety of water-soluble eggshell membrane hydrolysate using a dietary supplement (BiovaFlex, 450 mg daily) [70]. Researchers observed the knee function, mobility, and the overall health condition of 88 OA patients randomized into intervention (*n* = 44) or placebo (*n* = 44) groups. The clinical results were evaluated over a 12-week period. Compared to the placebo group, the patients with the poorest initial conditions benefited the most from the treatment with ESM hydrolysate with a significantly effect by Day 5, measured during the six-minute walk test. Other patients saw some obvious improvement by week 12 when compared with placebo group. Significant improvements were also seen from the normalized Stiffness score of Western Ontario McMaster Osteoarthritis Index by Day 5 [70]. These results indicated that the ESM hydrolysate can be used as a promising dietary supplement to relieve OA symptoms and enhance the mobility of OA patients. Similar conclusions have been presented in another double-blinded placebo-controlled ESM intervention trial. A total of 150 OA patients, between 45 and 70 years old, were randomly assigned to an intervention (*n* = 75) or placebo (*n* = 75) group [71]. After consuming 300 mg ESM powder on a daily basis for 12 weeks, patients self-reported that the treatment was successful in relieving the pain from their OA knee and contributed to improved daily life activities [71].

In addition to being used as a dietary supplement, ESM plays another important role in joint health. A study has shown that silk fibroin and polyvinyl alcohol with 3% autoclaved ESM presented similar magnitude of dynamic and compressive mechanical properties as the cartilage in human meniscus [72]. In addition, such scaffolding was beneficial to primary human meniscal cellular proliferation and extracellular matrix secretion. Researchers have discovered that ESM/silk fibroin hydrogels facilitated the adhesion and differentiation of human articular chondrocyte cells. Therefore, such hydrogels can be applied as cartilage substitute for tissue engineering in the future [73].

### 3.2. ESM for Wound Healing

ESM has been used as a biomaterial to promote the healing of skin wounds. Solubilized ESM may facilitate the synthesis of type III collagen in the skin of hairless mice as well as significantly improved the elasticity of human skin and reduced facial wrinkles [74].

Non-healing skin wounds are regarded as a major health problem globally, causing high morbidity and mortality. Processed eggshell membrane powder (PEP) offers great potential as a cost-effective wound-healing product. Using the mouse excisional wound-splinting model, researchers showed that PEP facilitated wound closure faster in the treated group than in the untreated groups [23,75]. Furthermore, sPEP stimulated matrix metalloproteinases (MMP) activities in both dermal fibroblasts and mouse skin during a 10-day incubation period. PEP also enhanced the MMP-2 protein levels and promoted the production of alpha-smooth muscle actin [76].

ESM patching may also offer a potential treatment for tympanic membrane (TM) perforation [77]. Researchers randomized traumatic TM perforation patients into two groups: the perforation edge approximation group and the eggshell membrane (ESM) patch group. The results showed that ESM patching significantly shortened the healing time, particularly in patients who were suffering from moderate to severe traumatic TM perforations [78].

Physicochemical properties of the ESM such as hydrophilicity and hardness can be modified using inorganic compounds. For instance, depositing copper (Cu)-containing bioactive glass nano-coatings (Cu-BG) on the ESM produced Cu-BG/ESM films that were able to significantly enhance angiogenesis in vivo, allowing the construction of constant and uniform epidermis layer, leading to higher healing quality. In addition, a substantial amount of Cu^2+^ ions released from these Cu-BG/ESM films significantly reduced bactericides and therefore prevented wound infection [79]. Researchers found that combining EMS with silver nanoparticles improved re-epithelialization, granulation tissue construction, and wound healing by facilitating cell proliferation and inhibiting inflammation [80].

### 3.3. ESM for Gut Health

The benefits of ESM have been shown in many reports addressing various gut-related diseases. In a murine model of dextran sodium sulfate-induced colitis, ESM powder was proved to suppress the disease activity index and colon shortening. It was shown to reduce intestinal inflammation by facilitating the restoration of the integrity of the epithelium and mitigating the effects of microbial dysbiosis [81]. In an in vitro study, ESM inhibited inflammatory cytokine production induced by lipopolysaccharide while ameliorated the Caco-2 cell proliferation by up-regulating growth factors. These effects were related to the significant improvements in gene expressions of inflammatory mediators, intestinal epithelial cell proliferation, restitution-related factors, and antimicrobial peptides [77]. By increasing the diversity of bacteria and decreasing the absolute numbers of pathogenic bacteria such as *Enterobacteriaceae* and *E. coli*, ESM plays an essential role in limiting dysbiosis. At the same time, ESM was also reported to regulate the expansion of Th17 cells by inhibiting the overgrowth of segmented filamentous bacteria. ESM supplementation in high-fat-diet-fed mice also decreased plasma triglycerides and liver total cholesterol by altering lipid metabolism gene expression and modifying the composition of the gut microbiota [82].

ESM hydrolysate also effectively suppressed pro-inflammatory cytokine IL-8 secretion in vitro and alleviated in vivo the signs of colitis induced by dextran sodium sulfate. Research demonstrated that ESM could relieve inflammation in a *colitic* mice model via the IL-6-mediated pathway and promotes T cells’ apoptosis to restore immune homeostasis in the gut [83].

### 3.4. ESM for Anti-Inflammatory and Antioxidant Activity

The anti-inflammatory effects of ESM have been also investigated in several studies. After being processed by cryo-grinding and homogenization into particles approaching submicron dimensions, ESM powder exerted some anti-inflammatory activity, while its antimicrobial activity against skin-associated pathogens was also enhanced [84]. The aqueous extract of ESM can also affect signaling events during responses to the T cell-specific mitogen phytohemagglutinin and pokeweed mitogen. This influence may be mediated through a decrease in the level of the pro-inflammatory cytokine TNF-α, providing some insights into the use of ESM as an anti-inflammatory product [85]. Vuong et al. also confirmed that both processed ESM power and EMS-derived soluble fractions demonstrated anti-inflammation and immunomodulation properties in lipopolysaccharide-triggered human monocytes and macrophage-like cells through the intervention of NF-κB [86]. Yoo et al. treated ESM with acetic acid and divided hydrolysate into fractions of different molecular weights. They found that the whole ESM hydrolysate and the fractions with more than 10 kDa presented some anti-lipopolysaccharide and anti-IFN-γ-induced inflammation activities as well as an outstanding effect on suppressing skin inflammation [87]. Ovocalyxin-36 is a protein from ESM with immuno-modulating effects. In vivo, peptides derived from ovocalyxin-36 are more effective at reducing LPS-induced inflammatory symptoms and inhibiting the local production of pro-inflammatory mediators in the small intestine [42]. Hence, ESM hydrolysates offer some promising leads as an oral anti-inflammatory product.

In addition, ESM hydrolysates prepared using a variety of alkaline proteases presented some excellent radical scavenging activity and protected the intestinal epithelial cells against oxidative stress induced by H_2_O_2_ [68]. The ESM hydrolysates prepared by a combination of Alcalase and Protease S were able to suppress the formation of H_2_O_2_-induced malondialdehyde and protein carbonyl. In addition, they improved the antioxidant enzyme activity and glutathione synthesis against oxidative damage in Caco-2 cells [88]. After fermentation with *Bacillus altitudinis*, lactic acid bacteria or other bacteria, ESM hydrolysates exhibited antioxidant and antihypertensive activities, preventing oxidative stress in vitro [89,90]. Further investigation found that the degree of hydrolysis of ESM hydrolysates showed a marked positive correlation with their antioxidant activity [91].

### 3.5. ESM for the Control of Bacteria

After modification by inorganic compounds, functionalized ESM exhibits a highly efficient antibacterial activity. For instance, copper-containing bioactive glass/eggshell membrane nanocomposites were able to maintain the sustained release of Cu^2+^ ions and showed marked antibacterial activity [79]. Various studies have shown that ESM with a series of silver nanoparticles (AgNPs) presented better antibacterial properties, suggesting that AgNPs/ESM composites may be potential antimicrobial product candidates for various therapeutic applications [80,92]. Preda et al. demonstrated that functionalized ESM combined with metal oxides CuO-ZnO showed powerful antibacterial activity against *Escherichia coli* when exposed to visible light due to an axial *p–n* junction [93]. Finally, the combination of ESM and chitosan in wound-dressing films was shown to greatly enhance their antibacterial activity [94].

### 3.6. ESM for Biomineralization

Biomineralization is a process in which specialized cells secrete and deliver inorganic ions into confined spaces within organic matrices or scaffolds. Calcitic biomineralization is essential in humans for the formation of otoconia, which is required to perceive linear acceleration and the effects of gravity. ESM can be applied as a biomineralization substitution of CaCO_3_ nano-crystals [95], with extracellular matrix (ECM) proteins from the ESM influencing the process of biomineralization. There are 46 proteins associated with the membrane fibers, and most of them are candidates for regulating calcitic biomineralization [26]. As a major component of the non-mineralized ESM, type X collagen has a controversial role in the biomineralization process. Some scientists reported that type X collagen suppresses cellular mineralization and limits the deposition of minerals [19], while others considered that it facilitates the regulation of calcification [96].

Recently, more studies focused on modifying ESM to serve as a biotemplate for crystal growth or as a biomineralization model. For example, ESM was a suitable biotemplate allowing hydroxyapatite crystals to form flower-like agglomerates [97]. ESM can also influence the type of CaCO_3_ polymorph during the initial stages of the repair process of the shell of the land snail *Helix aspersa* after an injury [98]. After treatment with sodium trimetaphosphate, phosphate groups were introduced onto the surface of type I collagen and strengthen the mineralization of ESM by forming calcium phosphate crystals [99]. In addition, it was shown that polycarboxylated ESM contained more surface nucleation sites for CaCO_3_ mineralization [100].

### 3.7. ESM for Immobilisation

ESM is not soluble in water, but it is permeable to water and air, making for its potential application as a biomaterial used for immobilization. ESM have proven to be efficient materials for the development of novel biosensors. Several researchers have shown the effectiveness of using ESM as a supporting matrix for the immobilization of enzymes such as urease, D-amino oxidase, catalase, myrosinase, tyrosinase, and glucose oxidase [101]. ESM treated with polyethyleneimine acquired polycation characteristics that were used for the immobilization of urease during the development of a potentiometric urea biosensor [102]. An amperometric horseradish peroxidase biosensor was also developed based on gold nanoparticles depositing on a three-dimensional porous carbonized ESM, proving to be remarkable for the detection of H_2_O_2_ detection both in terms of accuracy and sensitivity [103]. Relying on fluorescence resonance energy transfer, a potent acriflavine-immobilized ESM fluorescence biosensor was designed for the effective detection of Sudan I–IV, showing several advantages such as rare detection constraints, high sensitivity and selectivity, and perfect stability [103].

### 3.8. ESM for Tissue Engineering

ESM has been widely used as a low-cost and biodegradable natural material in tissue engineering applications. It has been used to develop different types of scaffolds for nerve tissue engineering that improved nerve regeneration [104,105]. Layered constructs from poly (ethylene glycol) hydrogels and ESM cross-linked by glutaraldehyde have shown heterogenic structures and mechanical properties comparable to heart valve leaflets, making them potential candidates for artificial heart valves replacement [106]. ESM powder (<100 μ in size) added into a collagen-based scaffold for 3D-tissue engineering improved the mechanical properties and the promotion of cellular adhesion and growth during cell regeneration [23]. ESM/thermoplastic polyurethane vascular graft with a wavy structure promoted endothelial cell proliferation by mimicking the vascular intima surface and reproducing the mechanical behavior of natural blood vessels [107].

### 3.9. ESM for Food Packaging

The edible films are safe and eco-friendly packaging materials to protect foods against oxygen, carbon dioxide, lipids, aroma, flavors, and moisture [108]. ESM as a food by-product contains abundant proteins, which has huge potential to be used in food packaging. The ESM-derived gelatin has been applied to produce edible films with chitosan. The addition of ESM in edible films showed that it could be an excellent material to improve the mechanical and barrier properties of films [109]. The SEP has been proved to interact with soybean protein isolate due to the hydrogen bonds. The protein-based composite film containing SEP, soy protein isolate, and eugenol showed the satisfying mechanical, barrier, water resistance, and hydrophobic features [110].

### 3.10. ESM for Biosorbent Activities

Due to its potential for chemical modifications, ESM is also an excellent biosorbent and is used to absorb various inorganic substances [111,112], dye [113,114,115,116,117], and other substances in aqueous solution.

The applications of the ESM along with the corresponding preparation methods are summarized in Table 2.

## 4. Safety Evaluation of Eggshell Membrane

ESM as a novel dietary ingredient has been evaluated for safety in a series of in vitro and in vivo studies. ESM-derived products have shown no cytotoxic effects at a dose of 100 μg in human cell viability assay after incubation for up to 20 h. ESM shows no genotoxic effects in a mutagenicity evaluation using histidine-dependent *Salmonella typhimurium* and tryptophan-dependent *Escherichia coli* at a dose of up to 5000 μg/plate. In animal studies, ESM did not exhibit any signs of acute toxicity after a single oral dose of up to 2000 mg/kg body weight. After the administration of repeated oral doses up to 2000 mg/kg body weight per day for 90 days, ESM did not cause any sign of toxicity as evaluated by urinalysis, hematology, clinical chemistry, or histopathological examinations [129]. The safety profile of ESM strengthens its potential as a candidate for various applications with the medical and food sectors.

## 5. Future Perspective

In addition to the applications presented previously, ESM offers more possible usages combined or not to the eggshell. Among the most promising applications, we can consider the use of ESM in electric devices. ESM is being investigated for the development of batteries as an alternative to lithium-ion. Carbonized ESM-based platforms are used for energy storage. Capacitors, electric components that are rapidly charged and discharged, are in high demand with the increasing use of portable devices. Studies have investigated the use of carbonised ESM for capacitors instead of carbon-based materials and conducting polymers. ESM is also suitable for the production of solar cells, semiconductors, and fuel cells [130].

## Figures and Tables

**Table 1:** Main chemical components of the eggshell membrane and their functions.

Main Components	Major Biochemical Functions
Collagens	- Optimum mechanical strength [23] - Thermal stability [23] - Wound healing [24] - Osteocompatibity [25] - Anchorage to nanohydroxyapatite [25] - Biomineralization [26]
Osteopontin	- Affinity binding for hydroxyapatite and osteoblasts [27,28] - Inhibitor of mineralization [28] - Modulation of osteoclast differentiation [29] - Recruitment of macrophages [30] - Regulation of cytokine production [30] - Inhibition of vascular calcification [30] - Regulation of apatite crystal size and growth [31] - Tissue remodeling [32]
Fibronectin	- Promotion of cell adhesion [33] - Improving cell growth, migration, and differentiation [33] - Wound healing [34]
Keratin	- Self-Assembly [35] - Promotion of cell adhesion [36]
Cysteine-rich eggshell membrane proteins (CREMPs)	- Wound healing [24]
Histones	- Chromatin folding and compaction [37] - Potent antimicrobial properties [38]
Avian beta defensins	- Promotion of innate defense system [39] - Reinforcement of the antimicrobial defenses associated with the ESM [40]
Ovocalyxin-36	- Potent antimicrobial properties [41] - Positive immune-modulating effects [42]
Apolipoproteins	- Binding and transport of lipids [43]
**Protocadherin**	- Adhesion and differentiation functions [44]
Chondroitin sulfate	- Formation of porous hydrated gels [45] - Immuno-inhibition property for articular cartilage repair [46] - Binding Ca^2+^ [47] - Molecules’ migration through the matrix [48]
Hyaluronic acid	- Water-retaining property [49] - Improving angiogenesis and tissue morphogenesis [50]

**Table 2:** Applications of ESM and its corresponding preparation methods.

Applications	Methods	Functions
Joint Health	- Partially hydrolyzed utilizing a gentle enzymatic process	- Improving recovery from exercise-induced joint pain and stiffness and also reduced the discomfort from stiffness [69]
	- ESM product suspended in 0.5% (*w*/*v*) methylcellulose in water	- Exhibiting beneficial effects on multiple indices of arthritis including inflammation, pannus, cartilage damage, bone resorption, and periosteal bone formation [118]
	- Fine ESM powder filled in gelatine capsules	- Promoting joint health and reducing pain and stiffness [71]
	- Soluble ESM treated by with aqueous 3-mercaptopropionic acid at 90 °C in presence of 10% (*v*/*v*) acetic acid	- Supporting growth, adhesion, and differentiation of human chondrocyte cells [73]
Wound healing	- Micronized ESM powder (<100 µm size)	- Improving wound closure through its structural ECM-like constituents that facilitated re-epithelialization [23] - Enhancing fibroblast and keratinocyte proliferation, myofibroblast differentiation, and regulation of the activity of various MMPs [76]
	- Round ESM patch with diameter of 5 mm	- Reducing healing time in patients with moderate to large traumatic tympanic membrane perforation [77]
	- ESM-chitosan blend film with 0.01 g ESM/mL 1% (*w*/*v*) chitosan solution	- Improving water resistance, wound fluid absorption, BSA absorption capacity, and antibacterial activity [94]
	- Incorporate AgNPs into ESM microfibers	- Accelerating wound healing with good biocompatibility [80]
	- Natural ESM	- Providing a scaffold for the fibroblast migration and reducing the lag phase for wound healing [119]
	- Copper-containing bioactive glass/ESM nanocomposites	- Enhancing angiogenesis-related gene expression as well as VEGF and HIF-1α protein secretion of HUVECs [79]
Immobilization	- ESM powder suspended in 50 mM sodium phosphate buffer (pH 7.0)	- Maintaining properties of β-galactosidase and allowing the reutilization of the immobilized enzyme to hydrolyze lactose in the presence of skim milk serum [120]
	- ESM/Tyr/AgNPs	- Platform for interference-free sensing of dopamine [121]
	- Small strips of ESM	- Use as energy-saving and biodegradable, laccase-based biocatalysts [122]
	- Natural ESM	- Excellent immobilized stability with a long shelf-life of bi-enzyme for highly-sensitive organophosphorus pesticide biosensors [123]
Antimicrobial activity	- Dried ESMs with dimensions of 2 cm × 3 cm functionalized only on one side with metal oxide or/and metal	- Antibacterial activity against *Escherichia coli* [93]
	- KR-12 peptide-containing hyaluronic acid immobilized fibrous ESM	- Good antibacterial activity against both Gram-negative and Gram-positive bacteria, including multi-drug-resistant bacteria [124]
Gut health	- Fine ESM powder	- Regulating the cell proliferation and restitution, improving energy metabolism as well as alleviating intestinal microbiota dysbiosis [81]
	- 8% ESM powder with corn starch and casein	- Altering lipid metabolism gene expression and gut microbiota composition [82]
	- ESM hydrolysate digested using a combination of Alcalase and Protease S	- Ameliorating intestinal inflammation induced by dextran sulfate sodium in mice [83]
Anti-inflammatory and antioxidant activity	- Micronized ESM powder (<100 µm size)	- Displaying anti-inflammatory activities through NF-κB in LPS-triggered human immune cells [86]
	- ESM dissolved in 2 N NaOH and 40% EtOH at 70 °C for 2 h	- Tyrosinase inhibiting and L-DOPA oxidizing activities [87]
	- ESM treated via in vitro digestion	- Regulating cytokine production in cultures of peripheral blood mononuclear cells and suppress tumor necrosis factor-α levels [85]
	- ESM fermented by lactic acid bacteria	- Inhibiting DPPH scavenging radical [89]
	- ESM dissolved in 1.25 M 3-mercaptopropionic acid and 10% acetic acid at 90 °C for 6 h and digested by 2% (*w*/*w*) pepsin at 37 °C for 4 h	- Inhibiting the H/R-induced and H_2_O_2_-challenged injury of cardiomyocytes and improving cardiac ischemia-reperfusion injury [125]
	- ESM treated by Na_2_SO_3_ and alkaline protease	- ABTS scavenging activity and liposomal peroxidation inhibitory activity [91]
	- ESM hydrolysate digested using a combination of Alcalase and Protease S	- Cellular antioxidant activity and protecting intestinal epithelial cells against oxidative stress [68]
Tissue engineering	- ESM tube conduit	- Enhancing peripheral nerve regeneration [105]
	- ESM powder below 0.5 µm size	- Increasing surface area of the scaffold, allowing better cellular infiltration and proliferation [72]
	- ESM guidance channel containing lycopene	- Increasing sciatic nerve regeneration [126]
	- ESM with dimension of 10 mm × 40 mm × 0.75 mm, soaked in the PCLF solution containing bisacylphosphinoxide	- Promoting the intercellular signaling leading to the enhanced cellular proliferation [104]
	- ESM soaked in 10% PCLF solution in acetic acid containing 5% bisacylphosphinoxide as the photo-initiator to make three-layered hollow tubular scaffold	- Enhancing nerve cell proliferation and orientation [105]
Food packaging	- ESM-derived gelatin-chitosan blend edible films	- Improving mechanical and barrier properties of films [109]
	- ESM powder mixed with soybean protein isolate, eugenol, and glycerol	- Enhancing the mechanical, barrier, water resistance, and hydrophobic properties [110]
Biosorbent	- ESM powder with size 0.5–0.6 mm	- Removing cyanide ions [127]
	- ESM powder immersed in methanol containing 2% (*v*/*v*) HCl for 10 h at 80 °C for carboxymethylation	- Removing anionic sulfur dye [128]
	- ESM powder with size 250–350 μm	- Removing organic cationic dye Basic Fuchsin [116]

## Data Availability

The datasets generated for this study are available on request to the corresponding author.
